# A144 CHRONIC DSS LEADS TO ALTERED EXPRESSION OF IRAK4/TPL2 PATHWAYS IN UC-COLONIZED MICE

**DOI:** 10.1093/jcag/gwab049.143

**Published:** 2022-02-21

**Authors:** A Hann, A Santiago, H J Galipeau, M Constante, K Jackson, P Bercik, E Verdu

**Affiliations:** 1 McMaster University, Hamilton, ON, Canada; 2 Medicine, McMaster University, Hamilton, ON, Canada

## Abstract

**Background:**

Ulcerative colitis (UC) is one of two forms of inflammatory bowel disease (IBD). The exact cause of IBD is unknown but altered host-microbe interactions and genetic susceptibility are involved in its pathogenesis. Many patients with IBD do not respond to biological therapies targeting single cytokines, therefore new therapies that target common immune pathways are being developed and need to be tested in relevant preclinical models. Previously we have shown that mice colonized with UC microbiota upregulated genes related to inflammation without induction of colitis compared to healthy volunteer-colonized mice. We thus investigated whether IRAK4 and TPL2-induced pathways, new therapeutic targets in development upstream of inflammatory cytokine gene activation, are upregulated in mice colonized with UC microbiota and chronic colitis.

**Aims:**

Our aim was to characterize TPL2 and IRAK4 signalling pathways and T cell phenotypes in UC-colonized mice following chronic low-dose dextran sodium sulfate (DSS) colitis.

**Methods:**

10-16-week-old germ-free C57BL/6 mice were colonized with fecal microbiota from a patient with UC experiencing a flare (n=16). Mice were housed in a gnotobiotic facility during the experiment. Three weeks following colonization, colitis was induced in half of the mice by three cycles (5 d each; 2.0%, 1.5% and 1.5%, respectively) of DSS in drinking water with a 5-d wash-out period between cycles. All mice were fed a control diet (7004, Teklad). Fecal samples were collected weekly. At sacrifice, disease activity (colon length, occult blood in feces, stool consistency, and spleen weight) was measured and colon tissue was collected for histological analysis and RNA sequencing. Mesenteric lymph nodes (MLNs) were acquired for flow cytometry to analyze T cell phenotypes.

**Results:**

In UC-colonized mice, chronic low-dose DSS induced softer feces (p<0.0001), shorter colon length (p<0.0001), and increased spleen weight (p<0.0001), compared with water treated mice. This was paralleled by detectable blood in stool, development of moderate colitis (DSS: 1.9+/-1.4 vs H_2_O: 0.5+/-0.2; p<0.02) and higher proportion of IL-17 (p=0.003) and IFN-γ-producing T cells (p=0.06) in MLNs compared with water treated mice. RNA sequencing revealed that inflammatory genes, mainly related to the IRAK4/TPL2 pathway (e.g., *Gadd45b*, *Socs3, Il1b*), were significantly increased (p≤0.05) in the colon of mice treated with DSS compared with water.

**Conclusions:**

When challenged with a chronic low-dose chemical injury agent, mice colonized with UC microbiota develop clinical and histological signs of colitis and upregulation of genes involved in inflammation like *Gadd45b*, Socs3, and *Il1b*. Thus, this model represents a new valuable tool for preclinical testing of new drug candidates, such as those related to the modulation of IRAK4/TPL2 pathways.

**Funding Agencies:**

CCC

